# Complications following Treatment of Trochanteric Fractures with the Gamma3 Nail: Is the Latest Version of Gamma Nail Superior to Its Predecessor?

**DOI:** 10.1155/2014/143598

**Published:** 2014-02-06

**Authors:** Dimitrios Georgiannos, Vasilios Lampridis, Ilias Bisbinas

**Affiliations:** ^1^424 Military General Training Hospital, PC, 56429 Thessaloniki, Greece; ^2^Royal Bournemouth Hospital, Castle Lane East, Bournemouth BH7 7DW, UK

## Abstract

Gamma nail is a cephalomedullary implant that was developed for the treatment of pertrochanteric hip fractures and has been successfully used for over 20 years. During this period, modifications of design and instrumentation have occurred to combat the intra- and postoperative complications that were associated with the use of early designs. The purpose of this study was to compare the complications observed with the use of the Gamma3 nail (G3N) with those seen following use of the previous trochanteric gamma nail (TGN). This study prospectively recorded the intra- and postoperative complications of 175 patients treated with the Gamma3 nail and compared them with those of a historical cohort of 192 patients treated with the trochanteric gamma nail. We encountered less intra- and postoperative complications with the use of Gamma3 nail. Femoral fractures and lag screw cutout were significantly lower. The reoperation rate was significantly higher in the TGN group. Gamma3 nail has proved to be a safe and efficient implant for the treatment of pertrochanteric fractures. The improvement of the biomechanical characteristics has led to a significant decrease in complication rates, demonstrating superiority over its predecessor.

## 1. Introduction

Fractures in the trochanteric region of the femur are very common in the elderly. The elderly population is increasing steadily making treatment of these fractures increasingly important in terms of medical, social, and economical issues.

Cephalomedullary nailing is theoretically the most stable and least invasive method of fixation. Biomechanical examinations had shown that intramedullary devices might be superior to plating systems, especially in unstable extracapsular fractures of the proximal femur [1]. The standard gamma nail (SGN) was the first intramedullary device introduced to provide a sliding cervical lag screw that would allow controlled fracture impaction and intramedullary fixation in the femoral shaft. It has also proven to be effective in the minimization of surgical trauma, blood loss, bone devascularisation, and wound complications [2]. But, clinically, the SGN was associated with a high rate of intra- and postoperative complications—in particular femoral fracture- and reoperation [2–4]. For that reason, modifications of design and instrumentation have occurred, resulting in the most recent version, the Gamma3 nail (G3N) (STRYKER Trauma GmbH, Schönkirchen, Germany).

The purpose of this study was to compare the complications of the treatment of trochanteric fractures with the G3N and the second version of gamma nail, the trochanteric gamma nail (TGN).

## 2. Method

The prospective study group consisted of patients that had been treated for trochanteric fractures with the G3N in the period between 2006 and 2009. The historical cohort consisted of patients that had been treated with the TGN between 2000 and 2005.

All operations were performed by four orthopaedic surgeons with global knowledge of the principles of intramedullary nailing and experience in the use of gamma nails, as gamma nail has been exclusively used in our department since the first generation—the SGN—was introduced. The method of treatment was similar to both groups. Patients were positioned supine in traction table and closed reduction of fracture obtained under fluoroscopic control. All intramedullary canals were reamed up to 13 mm distally for both nails and proximally up to 15.5 mm and to 17 mm for G3N and TGN, respectively. Lag screw was inserted in a 130° angle, optimally in apposition inferiorly to the neck in the AP plane and centrally in the lateral plane. All short nails were locked distally with one locking screw using the targeting device and all long nails were locked with two distal screws with a free hand technique. All patients mobilized fully weight bearing as tolerated at first postoperative day.

The following variables were collected: patients' age and gender, mechanism of injury, fracture type, waiting time to surgery, operation time, fluoroscopy time, duration of hospital stay, intra- and postoperative complications, and mortality rate. Patients were followed up at 6 weeks, 3 months, and 1 year with clinical and radiological assessment. Statistical analyses using the unpaired Student's *t* test and Fisher's exact test were applied to evaluate significant differences between the two groups (*a* was set at 0.05).

## 3. Results

Between 2006 and 2009, 175 patients were admitted in our department with an intertrochanteric and intersubtrochanteric fracture, treated surgically with a G3N (group A). We used 142 short (SG3N) and 33 long (LG3N) G3N. The historical cohort group (group B) consisted of 192 TGN—151 short (STGN) and 41 long (LTGN). Mortality rate at 1 year was 26.8% (47 patients) in group A and 26.5% (51 patients) in group B.

Preoperative patient data are shown in Table 1. The average age was 79 years (range 29–97 years) for group A and 81 years (range 48–96 years) for group B. The sex ratio between females and males was 2.6 : 1 and 2.8 : 1 for the two groups, respectively. Fractures were classified according to AO classification and the results for the two groups are shown in Table 1. Mechanism of injury was a simple fall in majority of cases (88% and 90%, resp.). Road traffic accident was responsible for 5% and 7% of fractures and a fall from height for 6% and 3% of fractures for the two groups, respectively.

Average waiting time to surgery was 24 hours (range 12–56 hours) for group A and 22 hours (range 12–50 hours) for group B. Average surgical time (skin to skin) was 38 min (range 17–62 min) and 43 min (range 17–84 min), respectively. Fluoroscopy time was 32 sec (range 21–65 sec) for group A and 45 sec (range 23–87 sec) for group B.

The differences between the two groups in the waiting time to surgery (*P* = 0.28) and the surgical time (*P* = 0.11) were not statistically significant. Fluoroscopy time in group A was statistically significantly reduced compared with group B (*P* < 0.001).

Intraoperative complications are presented in Table 2. Four complications in group A and 14 in group B were reported. The difference between the total number of intraoperative complications in the two groups was considered to be statistically significant (*P* = 0.04).

The major complications encountered with the use of TGN were 6 intraoperative fractures of femur. In 3 cases, the fracture was an undisplaced crack of the lateral cortex of the femoral shaft just distally to the tip of nail; these were treated conservatively with nonweight bearing mobilization until callus formation was seen radiographically. One case of shaft fracture was treated with internal fixation with cable-plate and two cases of greater trochanter fracture were treated with partial weight bearing mobilization for 6 weeks. There was significant difference between the two groups (*P* = 0.03); no femoral fractures were encountered in the G3N group.

Breakage of drill occurred in 1 and 4 cases of LG3N and LTGN, respectively, but no subsequent action was required. The broken drill bits were left in situ and the second distal screw inserted uneventfully. Open reduction was performed in 6 cases (2 in group A and 4 in group B). In 3 cases (2 in group A and 1 in group B) the fracture reduction was lost intraoperatively but no further action was taken due to critical medical problems of the patients. Perforation of acetabulum by the lag screw occurred in one case of G3N; the lag screw was revised with a shorter one.

Postoperative complications are presented in Table 3. We encountered in total 13 postoperative complications in group A (7.41%) and 30 in group B (15.62%). There was significant difference between the two groups (*P* = 0.03). The differences between the two groups for postoperative femoral fractures (*P* = 0.24), nail breakage (*P* = 0.49), distal screw breakage (*P* = 0.72), loss of reduction (*P* = 1.00), and nonunion (*P* = 0.72) were not significant. The difference in lag screw cutout complication was statistically significant (*P* = 0.04).

Femoral fracture occurred postoperatively in 3 patients of group B, following a fall. Two of those sustained a fracture just distal to the tip of the nail (one patient was treated conservatively and one patient was treated with an open reduction and internal fixation) and one patient sustained a neck of femur fracture which treated with a hemiarthroplasty after removal of nail.

**Figure 1:**
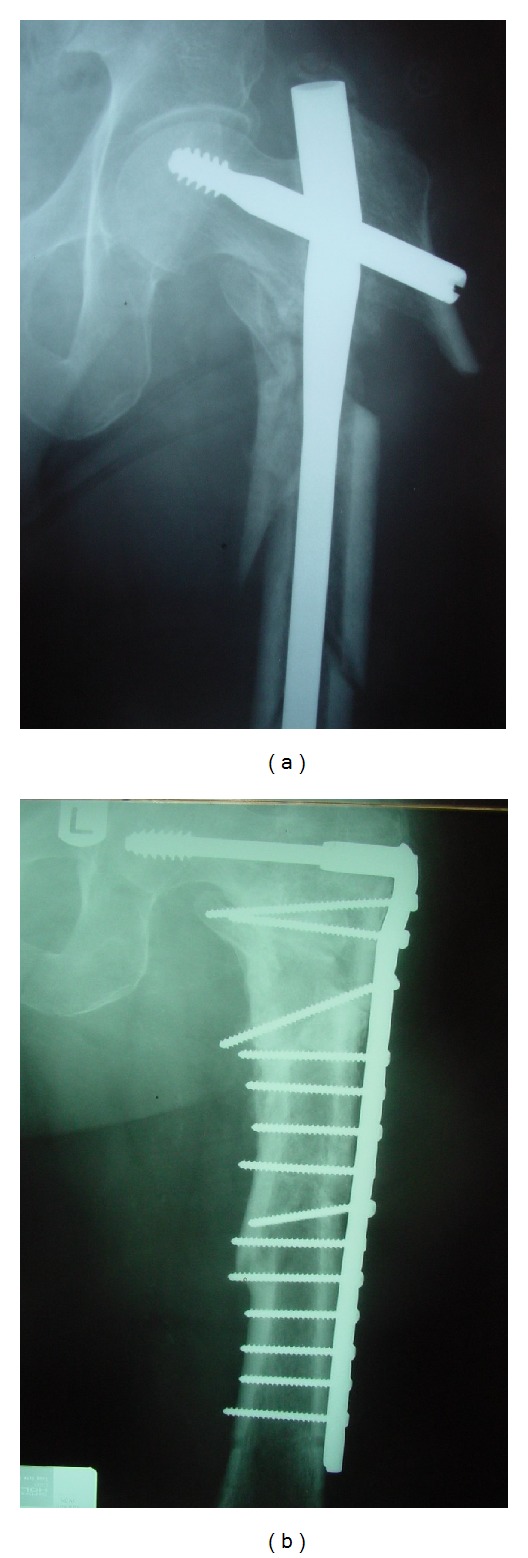
AP radiograph of a complex intersubtrochanteric fracture of femur, showing a broken long TGN at the junction of the nail with the lag screw (a). The nail was revised to DCS plate and the fracture healed at 4 months postoperatively (b).

In two cases, a TGN failed at the junction of nail with the lag screw, 4 and 6 months postoperatively, due to delayed union. The nails were revised to DCS and the fractures healed uneventfully 4 months after revision operation (Figure 1).

**Figure 2:**
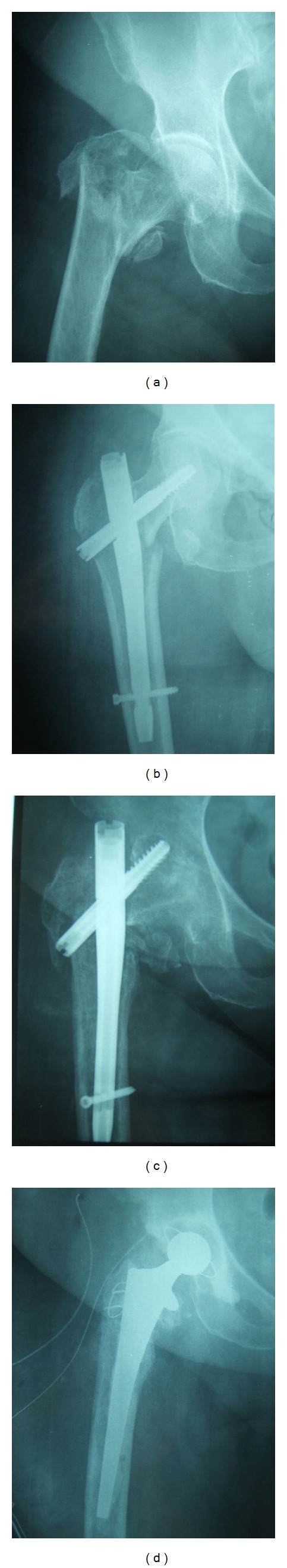
AP radiograph of an 81 yr patient with a 3-part intertrochanteric femoral fracture (a) treated with a short G3N (b). Cutout of the lag screw at 2 months postoperatively (c) treated with a THR (d).

The most frequent complication in both groups was the cutout of the lag screw (4 and 13 cases, resp.) which resulted in reoperation in 4 cases of group A (2 total hip replacements and 2 hemiarthroplasties) (Figure 2) and in 10 cases of group B (4 total hip replacements, 4 hemiarthroplasties, and 2 DCS).

In group A, loss of reduction occurred in 2 cases (treated with DCS) and nonunion in 3 cases of subtrochanteric fracture which were treated by revision to a long gamma nail with bone grafting. In group B, nonunion rate was higher (5 cases) and all were treated with revision nailing and bone grafting. Loss of reduction occurred in 3 cases; one case was revised with a DHS and two cases with a long gamma nail.

The overall reoperation rate was 5.71% (10 cases) for group A and 11.45% (22 cases) for group B, as it is shown in Table 4. The difference of reoperation rates between the two groups was significant (*P* = 0.04).

## 4. Discussion

Gamma nail is an implant that was developed for the treatment of trochanteric hip fractures and was first brought to use in 1988. The long gamma nail (LGN) was introduced in 1992 and was used for subtrochanteric and combined trochantero-diaphyseal fractures of the femur [5].

Biomechanical studies have shown advantages over extramedullary devices, combining a sliding lag screw for controlled fracture impaction and intramedullary fixation in the femoral shaft decreasing the bending moment arm of the loading forces on the implant by 25–30% as compared with extramedullary devices [6]. Despite these advantages, gamma nail has been historically related to devastating complications such as implant failure [7, 8] and femoral fractures [9], which eventually required revision surgery.

Trochanteric gamma nail (TGN) was introduced in 1997. Modifications, including reduced length from 200 mm to 180 mm, standard proximal diameter of 17 mm and distal diameter of 11 mm, and reduced mediolateral curvature from 10° to 4° [10], were the reasons of dramatic decrease of the rates of complications [11, 12]. Last modification of gamma nail is G3N which was introduced in 2003. In comparison with the TGN, it is narrower proximally (15.5 mm) and has also got a mediolateral curvature of 4° but with its apex positioned more distally. 5 mm screws are used for distal locking. The lag screw shape has been improved, especially in the area of the thread and the cutting flutes at the tip of the screw. Long G3N has got the same technical characteristics as the short G3N and has an antecurvature radius *R* 2.0 m of the femoral shaft, which is more anatomical compared with the *R* 3.0 m curvature of the TGN.

Only few studies have been published until today regarding G3N; the new design seems superior to previous generations, giving promising outcomes and reduced mechanical complication rates. Fracture of the femoral shaft at the tip of the nail is a known complication associated with the use of a gamma nail in the treatment of proximal femoral fractures. The SGN had a mediolateral curvature of 10° that differed from the trochanter-to-diaphysis angle in an average patient. This shape of the gamma nail is thought to cause three-point loading across the trochanter and diaphyseal cortices. Therefore, stress is concentrated mainly along the medial cortex in contact with the nail curvature and on the nail tip in contact with the lateral cortex, thus exposing the femur to intraoperative and postoperative fractures, even under physiologic load [13]. Results from other studies show high numbers of femoral shaft fractures, up to 17% for SGN [4, 9, 14] and up to 4.5% for TGN [5, 11, 12, 15–17]. In our study, 9 femoral fractures (4.68%) occurred in our historical cohort of TGN (6 intraoperatively and 3 postoperatively). No fracture of femur occurred in the G3N group of patients, which is similar to the results of other studies on G3N which is less than 1% [18, 19]. We attribute the low rate of the femoral shaft fractures to improvement of mechanical characteristics of the new design, namely, the decreased proximal diameter which requires less reaming and the distally positioned apex of the mediolateral curvature of the nail which reduces the three-point loading at the femoral shaft [10].

Breakage of gamma nail occurred at the junction of the nail with the lag screw and was reported in the literature in an incidence of up to 5.7% [7, 8, 13, 20]. In our study, none of the G3N failed, in contrast with 2 TGN broken nails (1.1%). It is known that the weak point of this implant was around the insertion hole for the lag screw where the cross-sectional area was reduced by approximately 73%. This is a critical zone where forces coming from the femoral neck are transmitted to the diaphyseal nail [7]. We believed that the decreased incidence of failure of the nail was attributed to the reduction of the lag screw diameter from 12 mm to 10.5 mm. Therefore, the aperture is smaller and thus the nail would be thicker in this area and less prone to failure. Delayed union/nonunion at the fracture site was the trigger factor for both the implant failures. The cause of breakage was metal fatigue due to dynamic stress [8, 21].

The most frequently occurring complication was the cutout of the lag screw through the femoral head (2.28% versus 6.77%). Our results were similar to the results of other studies showing an incidence rate of up to 9.72% [5, 10, 12, 17, 22] for the TGN and up to 4% for G3N [18]. Lag screw cutout has been shown to be dependent on the position of the screw within the femoral head. Optimizing tip-apex distance is critical in preventing fixation failure when using an extramedullary sliding hip screw to fix pertrochanteric fractures [23]. A recent study suggests that placement of the lag screw of the gamma nail inferiorly in the AP plane and centrally in the lateral plane maximizes biomechanical stiffness and load-to-failure of the fixation [24]. The position of the lag screw was considered optimal (inferiorly in AP/centrally in lateral plane) in 2 out of the 4 failed cases in group A and in 6 out of the 13 failed cases in group B. In the remainder of the failed cases, the position was considered suboptimal (centrally in AP/centrally or anteriorly in lateral plane). Therefore, we attributed the lower rate of cutout complication to the improvement of lag screw design, especially in the area of the thread and the cutting flutes at the tip of the screw. This design offers superior cutting behavior during lag screw insertion, providing very low insertion torque. The thread design also offers excellent grip in the cancellous bone of the femoral head and strong resistance against cutout.

The rate of reoperation after complications with the G3N was 5.11%, which was similar to the 5.56% rate reported in another study [25]. The rate of implant-related complications that required reoperation after primary use of the TGN was 11.45%. It is also in accordance with previously reported results ranging from 8% to 16.6% [16, 17, 22, 26].

The main limitation of this study is the use of a historical cohort as the control group. But the fact that this study focused mainly on implant-related complications and all operations were performed by the same group of experienced surgeons, we believe that increases the strength of the study and overruns the limitation of the use of a historical group. The second limitation is the number of patients that withdrew before the final follow-up at one year. Many patients had concomitant illnesses affecting their general health, making it impossible to participate in follow-up. Dropout rate was comparable between the two groups, leading to no bias in the interpretation of the results.

## 5. Conclusion

Within the limits of this study, Gamma3 nail has proved to be a safe and efficient implant for the treatment of pertrochanteric fractures. The improvement of its biomechanical characteristics has led to a significant decrease in observed complication rates, demonstrating superiority over its predecessor.

## Figures and Tables

**Table 1:** Preoperative patient data.

Data	Group A	Group B
Number of patients	175	192
Age	79 (29–97)	81 (48–96)
Gender		
(F/M ratio)	2.6 : 1	2.8 : 1
Classification		
31 A1	52	54
31 A2	60	68
31 A3	18	21
31 B2	19	18
32 A	16	17
32 B	10	14
Mechanism of injury		
Simple fall	88%	90%
RTA	5%	7%
Fall from height	6%	3%

**Table 2:** Intraoperative complications.

Complications	Group A (*n* = 175)	Group B (*n* = 192)	*P* value
Femoral fracture	—	6	0.03∗
Breakage of drill	1	4	0.37
Reduction difficulties—open reduction	2	4	0.68
Perforation of acetabulum	1	—	

Total	4 (2.28%)	14 (7.29%)	0.04∗

*Statistically significant.

**Table 3:** Postoperative complications.

Complications	Group A (*n* = 175)		Group B (*n* = 192)		*P* value
Femoral fracture	—	—	3	1.56%	0.24
Nail breakage	—	—	2	1.04%	0.49
Lag screw cutout	4	2.28%	13	6.77%	0.04∗
Distal screw breakage	4	2.28%	4	2.08%	0.72
Loss of reduction	2	1.14%	3	1.56%	1.00
Nonunion	3	1.71%	5	2.60%	0.72

Total	13	7.41%	30	15.62%	0.03∗

*Statistically significant.

**Table 4:** Reoperation data.

Data	Femoral fracture	Implant failure	Lag screw cutout	Loss of reduction	Nonunion
Group A (*n* = 9) (5.14%)	—	—	4 2 THA 2 Hemi	2 DCS	3 LGN and graft
Group B (*n* = 22) (11.45%)	2 1 ORIF 1 Hemi	2 revision DCS	10 4 THA 4 Hemi 2DCS	3 2 LGN 1 DHS	5 4 LGN and graft 1 LGN

*P* value: 0.04 (statistically significant).
